# Metabolic consequences of perinatal bisphenol A and 17α-Ethinylestradiol exposure manifest in circadian alterations of energy homeostasis in adult male mice

**DOI:** 10.3389/fendo.2025.1706909

**Published:** 2026-01-06

**Authors:** Imre Kalló, Andrea Kádár, Barbara Göblyös, Csaba Vastagh, Dániel M. Pap, Csaba Fekete, Zsolt Liposits

**Affiliations:** 1 Laboratory of Endocrine Neurobiology, Hungarian Research Network (HUN-REN) Institute of Experimental Medicine, Budapest, Hungary; 2 Laboratory of Integrative Neuroendocrinology, HUN-REN Institute of Experimental Medicine, Budapest, Hungary; 3 Roska Tamás Doctoral School of Sciences and Technology, Faculty of Information Technology and Bionics, Pázmány Péter Catholic University, Budapest, Hungary; 4 Laboratory of Reproductive Neurobiology, HUN-REN Institute of Experimental Medicine, Budapest, Hungary

**Keywords:** bisphenol A, ethinylestradiol, circadian rhythm, food intake, energy homeostasis, locomotion, endocrine disruptor

## Abstract

**Introduction:**

Environmental estrogenic chemicals can cross the maternal–fetal barrier and disrupt endocrine and metabolic regulation in the developing embryo/fetus. Bisphenol A (BPA) and 17α-ethinylestradiol (EE2) are widely present in the environment and have been linked to increased cardio-metabolic disease risk.

**Purpose:**

This study investigated the effects of maternal BPA and EE2 exposure on metabolic function and circadian energy regulation in male offspring.

**Methods:**

Pregnant and lactating dams were chronically administered BPA (20 µg/kg bw/day) or EE2 (0.01 µg/kg bw/day) via osmotic minipumps from gestational day 9 to postnatal day 21 to mimic environmental exposure. Adult male offspring (60–80 days old) were assessed for body composition, fasting glucose, and metabolic and activity parameters using the TSE Phenomaster system.

**Results:**

BPA-exposed offspring exhibited reduced lean body mass, fat mass, fat ratio, and 24-hour fasting glucose levels compared to controls and EE2-exposed offspring. Both BPA- and EE2-exposed groups showed altered circadian patterns of locomotor activity, food intake, energy expenditure, and respiratory exchange ratio, with effects predominantly occurring during the night phase.

**Conclusions:**

Maternal exposure to environmentally relevant doses of BPA or EE2 can alter the development and function of metabolic regulatory systems, producing distinct disruptions in circadian energy homeostasis in adult offspring. These differential effects likely reflect the partially overlapping yet distinct organizational and activational pathways through which these endocrine-disrupting chemicals act during the perinatal period.

## 1 Introduction

Metabolic functions are evolutionarily adapted to circadian changes in food availability, leading to species-specific phase preferences (diurnal or nocturnal) that ensure optimal environmental and social conditions for these physiological processes. Endogenous estrogens and estrogenic metabolites play critical organizational and activational roles in the reproductive system. They also regulate metabolic neuronal circuits and processes that support successful reproduction (1). It is concerning, however, that environmental chemicals with estrogenic effects - such as bisphenol A (BPA) and 17α-Ethinylestradiol (EE2) - can interfere with natural estrogenic signaling (2), potentially causing subtle but impactful changes in metabolism and reproductive health in affected species, including humans. Although recent European regulations have imposed strict bans on the use of BPA-releasing materials in the food industry (3), and BPA use in children’s products has been largely discontinued worldwide, human exposure persists. This is due to the slow phase-out of legacy products such as resin-coated drink containers, water pipelines, and dental materials (4). Similarly, environmental exposure to derivatives of natural and pharmaceutical estrogens is expected to continue in the coming years, primarily via diet (5). Pregnant and lactating women and their infants are particularly vulnerable. Both BPA and EE2 can cross the maternal–fetal barrier, and their parent compounds and metabolites have been detected in breast milk. BPA clearance is slower in the fetus than in the maternal circulation (6). Therefore, even low maternal doses of these chemicals may pose developmental risks for the offspring (7), especially during critical prenatal and early postnatal periods (8). Their action is further enhanced by the inability of alpha-fetoprotein to bind these compounds (9, 10).

Over the past few decades, the global incidence of metabolic diseases has surged. However, the potential etiological contribution of increasing environmental exposure to BPA and EE2 remains unclear. Previous reports have indicated that unlike females - where a clearer consensus is emerging on the diet-dependent metabolic effects of estrogenic pollutants (11–13) - male offspring often show inconsistent metabolic outcomes following perinatal BPA exposure. Across studies, body mass has been reported as increased (11, 12), unchanged (13–16), or decreased (17–20), and fat mass as increased (12, 15, 21, 22) or unchanged (13, 23, 24), with little evidence for altered food intake (25). Similarly, fasting blood glucose levels have been reported as either elevated (11) or unchanged (26). This sexual dimorphism is somewhat surprising, as fetal BPA concentrations are typically higher in males than in females (27). Nevertheless, male offspring appear less metabolically responsive to early-life exposure to estrogenic endocrine disruptors. Some effects may nevertheless go undetected when only basic metabolic variables (e.g., body weight, fat mass, fasting glucose) or organ-specific endpoints are assessed (14).

To address these limitations, the present study used the TSE PhenoMaster metabolic cage system, which enables high-resolution, continuous monitoring of energy intake and expenditure, allowing us to detect subtle or latent effects of perinatal BPA and EE2 exposure on the circadian regulation of metabolism. We also included a parallel group exposed to the estrogenic comparator EE2 to improve interpretability.

Thus, the present study focuses on the circadian patterns of food and water intake, locomotor activity, energy expenditure, and respiratory exchange ratio in young adult F1 male offspring exposed to BPA and EE2 in the sensitive perinatal period.

## 2 Materials and methods

### 2.1 Animals - mothers

Young (80–120 day old) female (n=50) and male (n=50) mice expressing enhanced green fluorescent protein (eGFP) under the control of GnRH promoter (15), were housed in BPA-free cages (polysulfone, PSU) under controlled humidity and light-dark conditions (light on 6:00 **–** Zeitgeber time (ZT) 0, light off 18:00 – ZT12). Water (in PSU bottles) and food (Ssniff S8189-S095 **–** standard breeding chow, **Supplementary File 1**) were provided *ad libitum.*

Female mice were then paired with sexually experienced male mice and observed for pregnancy for two weeks. Pregnant females were selected and kept with males until postdelivery days 2 or three, when dams are normally fertilized again (16). The vaginal opening of the dams was checked for plug during this period to determine gestational day 1 (GD1). Pups of the first breeding were eliminated from the litter during the 5-6th GDs.

During the second breeding, dams were briefly anesthetized on the 9^th^ GD using isoflurane, and implanted subcutaneously with osmotic minipumps (Alzet, Mini-Osmotic Pump, Model 2006; 15 µl/h, 42 days) through a small cut of the back skin. Pumps were filled with a DMSO/propylene glycol solution (vehicle) containing either BPA or EE2 in a concentration capable of delivering 20 µg/kg body weight (bw)/day or 0.01 µg/kg bw/day, respectively. Vehicle- (n=4), BPA- (n=6) and EE2-(n=4) treated mothers gave birth to 14, 21 and 17 male pups, respectively. Birth of the male and female pups were recorded; they were kept together until weaning.

### 2.2 Animals - offspring

Male offspring were caged after weaning in small groups (animals from the same litter were kept together until metabolic examination, **Supplementary File 2**/**Worksheet 1**) and upon reaching the young adult age (60–80 day old) they were investigated for basic metabolic parameters i.e. total and lean body mass, food and water consumption, as well as glucose levels after 24h fasting. Their food and water intake, oxygen consumption and CO2 production (respiratory exchange ratio) and locomotor activity were monitored during the circadian day to reveal potential alteration in these parameters compared to the offspring of vehicle-treated mothers. Body composition was determined by EchoMRI (700 Whole Body Composition Analyzer; E26-233RM, Echo Medical Systems, Houston, TX, USA). After acclimatization to single housing (1 week) and training boxes (3 days), all experimental mice were transferred to metabolic (calo) cages for metabolic measurement. Data of metabolic- and activity variables (food- and O2 consumption, CO2 production and X-Y-Z locomotor activity) were automatically collected and summed up every 15 minutes in TSE Phenomaster system for 40 hours involving a single light- and two dark-phases of the circadian day. Energy expenditure (EE (kcal/h)) was calculated using the TSE Labmaster System software based on the Weir equation supplied by (EE = [3.941 (VO2) + 1.106 (VCO2)] x 1.44) (17). The estimated resting energy expenditure was calculated by selecting 15 minutes cycles preceded by one hour periods, when animals were eating less than 0.1 g chow and within this periods the last half an hour was spent with minimum locomotor activity (the XY and Z direction movements were less than 1% of the maximum activity observed in any of the 15 minute cycle during the recording time). The respiratory exchange ratio (RER) was calculated with the following formula: VCO2/VO2. Animals were then taken out of the metabolic cages and were fed ad libitum for 60 hours before fasting. After replacing corncob bedding with fresh, food-free, low-calorie bedding, food was withdrawn from the animals for 24 hours. A few droplets of blood were collected from the tail vein between ZT2 and ZT3 for measuring blood sugar levels using an Accu- Chek Aviva (Roche Diagnostic, UK).

### 2.3 Statistical analysis

During statistical analyses, individual offspring were treated as experimental units. To verify whether littermates could indeed be considered statistically independent, the potential litter effect was evaluated for each metabolic parameter and treatment group.

For this purpose, intra-class correlation coefficients (ICCs) were calculated using intercept-only mixed models (parameter ~ 1 + (1 | litter)) to quantify the proportion of variance attributable to within-litter similarity. Restricted likelihood ratio tests (RLRTs) were also performed to determine whether inclusion of the random effect (litter) significantly improved model fit. Parameters showing ICC values below 0.3 and/or non-significant random effects (p > 0.05) were considered independent of litter membership. In most cases, these criteria were met; when not, analyses were repeated using litter as experimental units. Detailed results of these assessments are presented in the **Supplementary File 2**/**Worksheets 2, 3**.

Comparisons between treatment groups (both at the individual and litter levels) were performed for various metabolic and physiological parameters (**Supplementary File 2**/**Worksheets 4–9**) using appropriate non-parametric statistical tests, as detailed below.

Differences in basic body composition parameters (lean body mass, fat mass, and fat ratio) and 24-hour fasting blood glucose levels between treatment groups were evaluated using the Kruskal–Wallis test. To characterize metabolism in the different experimental groups, the following parameters were analyzed: food and water consumption, locomotion, energy expenditure, and respiratory exchange ratio (RER). As these parameters exhibit circadian rhythms, data were collected every 15 minutes for more than one light and two dark phases, and average hourly data were used for further analyses. For RER and energy expenditure, the effects of treatment, Zeitgeber (ZT) time, and their interaction were tested using the non-parametric Scheirer–Ray–Hare test (a non-parametric alternative to two-way ANOVA). As the main effects were significant, Kruskal–Wallis tests were applied at each time point to compare treatment groups. For food intake and rearing activity, cumulative data were analyzed. The area under the curve (AUC) was calculated individually for each subject to assess treatment effects using Kruskal–Wallis tests. Both analyses were followed by Dunn’s *post hoc* tests to determine which treatment groups differed from each other significantly. Benjamini–Hochberg correction was applied for multiple comparisons.

To assess circadian variation in metabolic parameters within each treatment group, Friedman tests (non-parametric alternative to repeated-measures ANOVA) were used to compare the first night, first day, and second night. When significant, *post hoc* pairwise comparisons were performed using the Nemenyi test. Differences in resting energy expenditure between treatment groups during the day and night were evaluated using Kruskal–Wallis tests, followed by Dunn’s *post hoc* tests with Benjamini–Hochberg correction.

Data are presented as box plots (median, interquartile range, and outliers), or as mean ± SEM. Differences were considered statistically significant at p < 0.05.

## 3 Results

### 3.1 Chronic exposure of mothers to BPA leads to altered body composition of offspring

**Figure 1:**
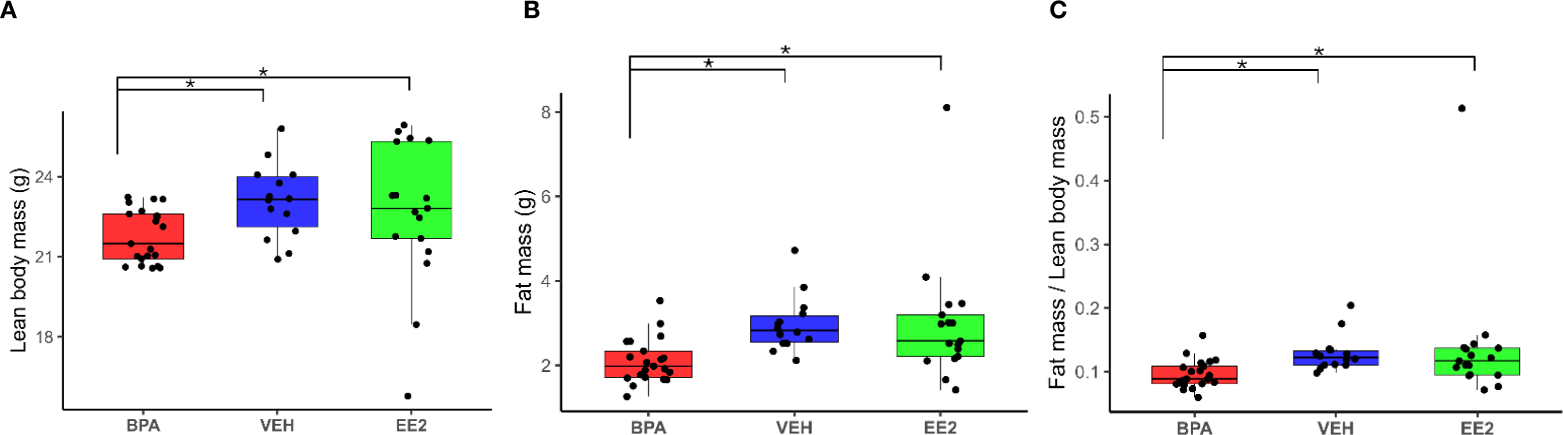
**(A–C)** Body composition of BPA offspring (n = 21) shows significant alterations compared with VEH (n = 14) and EE2 (n = 17) groups. Box plots display the median, interquartile range (25th–75th percentiles), and outliers for lean body mass, fat mass, and the fat mass-to-lean body mass ratio in each treatment group. Points represent individual mice. The reduced fat mass-to-lean body mass ratio indicates a lean phenotype in BPA-treated animals. Asterisks label significant differences. FM, fat mass; LBM, lean body mass; BPA, Bisphenol A; EE2, 17α-Ethinylestradiol; VEH, vehicle.

Analyses of the body composition using EchoMRI, revealed that BPA offspring show reduced lean body mass (p*_BPA-EE2_ = 0.0323* p*_BPA-VEH_ = 0.0238)* and fat mass (p*_BPA-EE2_ = 0.00764, p_BPA-VEH_ = 0.00116)* compared to VEH and EE2 offspring (*BPA_LBM_ 21.77 ± 0.22 mg; VEH_LBM_ 23.08 ± 0.37 mg; EE2_LBM_ 22.65 ± 0.65 mg)* (**Figure 1**). The fat ratio in BPA animals was also significantly lower *(BPA_F/TBM_ 0.087 ± 0.004; VEH_F/TBM_ 0.118 ± 0.008; EE2_F/TBM_ 0.103 ± 0.005/BPA_F/LBM_ 0.096 ± 0.005; VEH_F/LBM_ 0.129 ± 0.008; EE2_F/LBM_ 0.115 ± 0.006)* than in the VEH or EE2 mice (p*_BPA-EE2_ = 0,0138, p_BPA-VEH_ = 0,00188)* (**Figure 1**).

### 3.2 Circadian alterations in the activity and metabolic parameters of the animals

The locomotor activity and the energy expenditure of the experimental animals followed the circadian pattern of food and water intake of the animals. Thus, significantly increased values were detected in all experimental groups during the dark phase (night1-day2 differences/food intake: p < 0.001; water intake: p < 0.001; X-Y movement: p < 0.01; Z movement: p < 0.01; day2-night2 differences/food intake: p < 0.001; water intake: p < 0.001; X-Y movement: p < 0.05; Z movement: p < 0.001; exhibiting two peak values for each behavior at ZT13 and ZT19. A nadir appeared in all parameters at ZT6–8 during the light phase of the cycle. There were no significant differences between the night1-night2 values.

#### 3.2.1 Reduced food intake and unaltered water consumption characterize both the BPA and EE2 offspring

**Figure 2:**
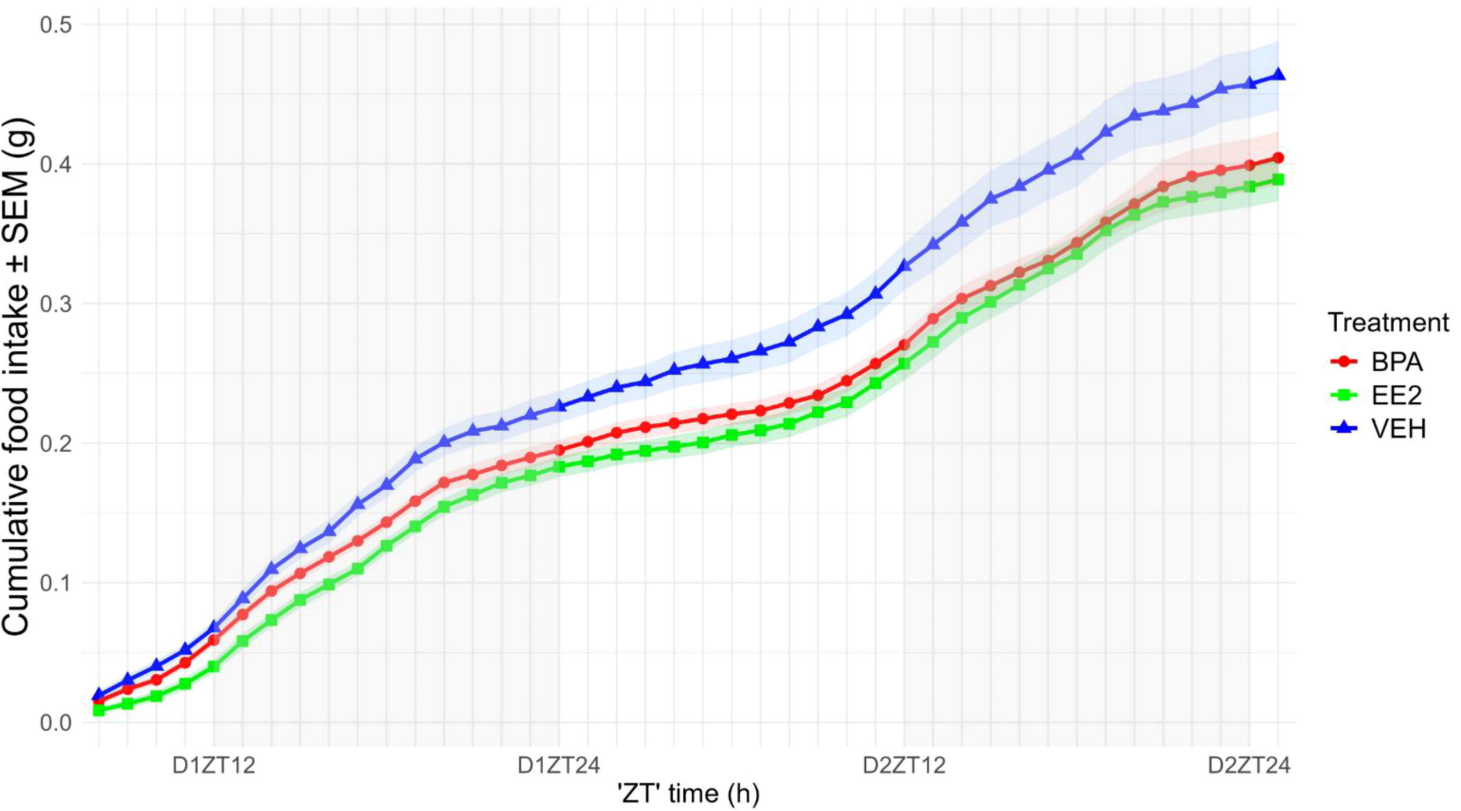
Cumulative food intake of the offspring (normalized to lean body mass; LBM) was recorded over 42 hours. BPA- and EE2-exposed offspring showed reduced food consumption compared with VEH controls. Data are plotted as group means ± SEM. over time (recording started at 2:00 p.m., corresponding to ZT8); grey bars indicate the dark phase (ZT12–ZT24). Lines and ribbons represent the group means and SEM, respectively. D1ZT12; Day1 Zeitgeber Time 12; BPA, Bisphenol A; EE2, 17α-Ethinylestradiol; VEH, vehicle.

To compare the effects of chronic BPA or EE2 treatment of mothers on the quantity of food consumed by the offspring, we measured the cumulative food intake of these animals and found a significant reduction in that for both the BPA (p < 0.05) and EE2 (p < 0.001) mice (**Figure 2** see also Image 2, as **Supplementary Figure 1** for values not corrected for lean body mass). VEH mice were eating more, which appeared as additional bouts of food consumption during the night phases of data recording. By analyzing the circadian pattern of food consumption, BPA offspring showed periods with reduced hourly food intake both during the active and the rest phases of the day, leading to a significantly lower food consumption in each phase (p < 0.05). The lower food consumption of EE2 offspring reached the significance level only for the light phase of the cycle (p < 0.01). No significant alteration could be observed in the water consumption of the different experimental groups (data not sown).

#### 3.2.2 Preferential Z-direction increase in the locomotor activity of BPA offspring

**Figure 3:**
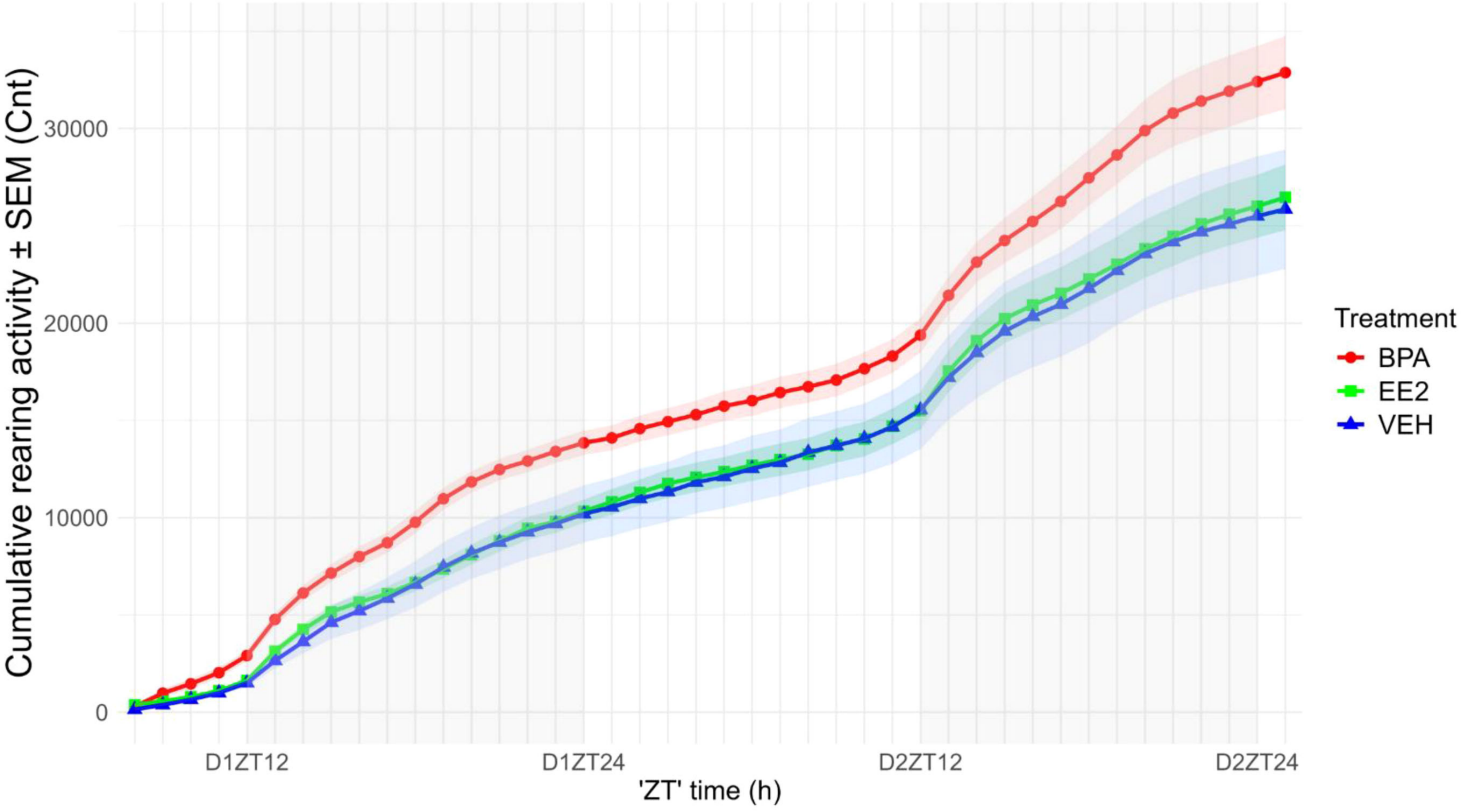
Cumulative rearing activity of the offspring was recorded by counting beam breaks over time. BPA-exposed offspring exhibited increased Z-direction activity compared with EE2 and VEH groups. Data are plotted as group means ± SEM. over time (recording started at 2:00 p.m., corresponding to ZT8); grey bars indicate the dark phase (ZT12–ZT24). The variation in rearing activity (width of the ribbons) appears reduced in offspring perinatally exposed to BPA or EE2. D1ZT12; Day1 Zeitgeber Time 12; BPA, Bisphenol A; EE2, 17α-Ethinylestradiol; VEH, vehicle.

As expected, there was a clear circadian influence on the X-Y direction locomotor activity displaying longer distances taken during the night than during the day, but there was no significant difference among the performances of the different experimental groups. In contrast, the rearing activity (Z direction movement) of BPA animals was significantly increased (p < 0.01), manifested with increased peaks at the onset of night and ZT18–19 during the night phases of the cycle. Unlike the EE2 offspring, which showed similar rearing activity in both phases as VEH offspring (**Figure 3**).

#### 3.2.3 A differential change in energy expenditure (EE) manifests in the BPA and EE2 offspring

**Figure 4:**
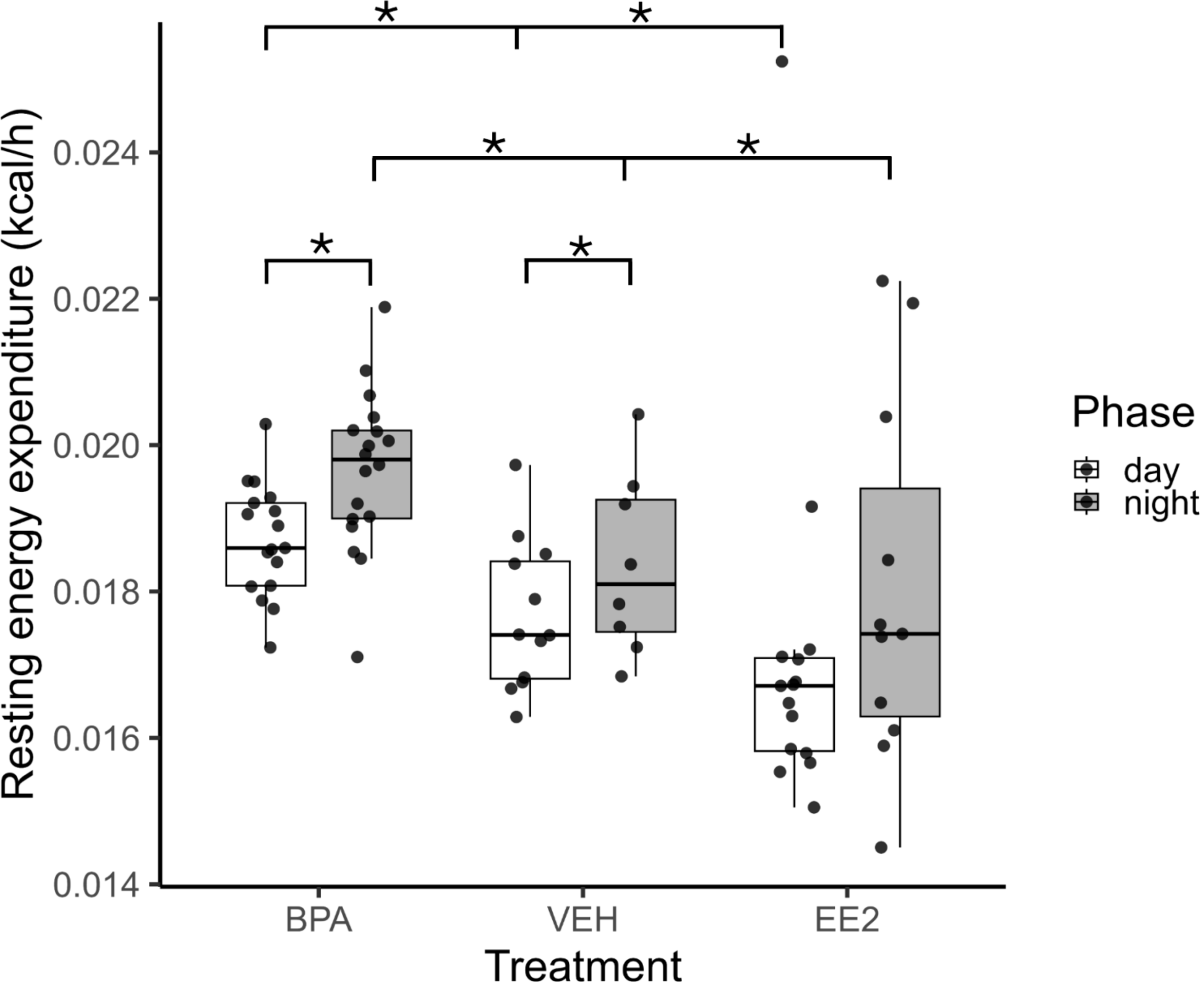
Estimated resting energy expenditure was calculated separately for the daytime (white columns) and nighttime (grey columns) periods in BPA, EE2, and VEH mice. Box plots display the median, interquartile range (25th–75th percentiles), and outliers for estimated resting energy expenditure within each treatment group, with points indicating individual mice. BPA- and EE2-exposed mice show differences in both phases compared with each other and with controls. All values were normalized to lean body mass (LBM). Asterisks label significant differences. BPA, Bisphenol A; EE2, 17α-Ethinylestradiol; VEH, vehicle.

**Figure 5:**
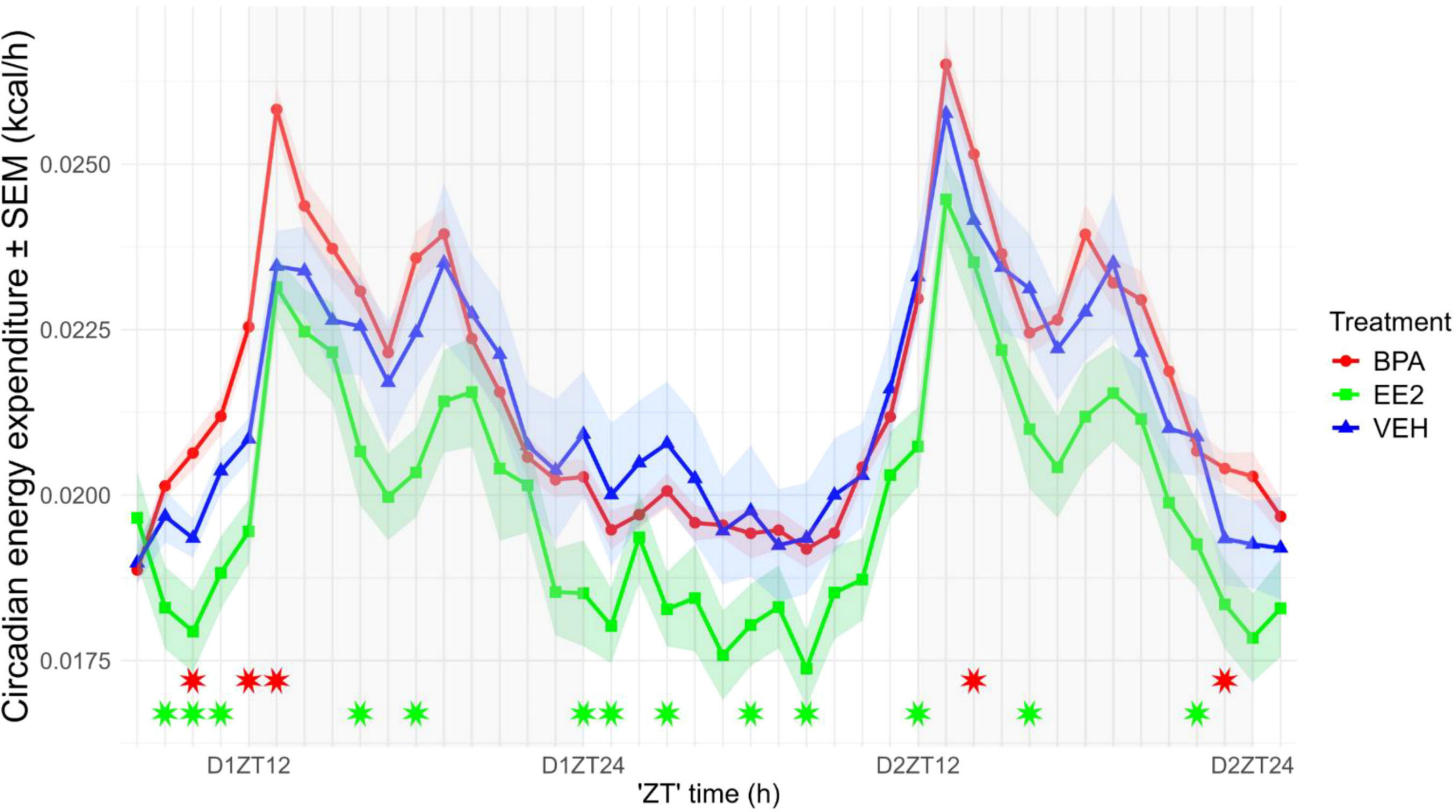
Circadian alterations in energy expenditure of the offspring (values normalized to lean body mass, LBM). Energy expenditure in EE2-exposed offspring was reduced throughout the circadian cycle. Data are plotted as group means ± SEM. over time (recording started at 2:00 p.m., corresponding to ZT8). Lines and ribbons represent the group means and SEM, respectively. Red (BPA) and green (EE2) asterisks indicate ZT time points at which values differ significantly from those of VEH offspring. D1ZT12; Day1 Zeitgeber Time 12; BPA, Bisphenol A; EE2, 17α-Ethinylestradiol; VEH, vehicle.

The resting energy expenditure (EE) of BPA-exposed mice (daytime: 0.01882 ± 0.0010 kcal/h/LBM; nighttime: 0.01972 ± 0.0017 kcal/h/LBM) was significantly elevated during both the day (p = 0.001) and night (p = 0.01), whereas EE2-exposed mice (daytime: 0.01692 ± 0.0002 kcal/h/LBM; nighttime: 0.0168 ± 0.0004 kcal/h/LBM) showed reduced values in both phases (p < 0.001; p < 0.01) compared with VEH controls (daytime: 0.0179 ± 0.0002 kcal/h/LBM; nighttime: 0.0187 ± 0.0003 kcal/h/LBM). The day–night difference in resting EE was preserved in BPA animals but was abolished in EE2 mice (**Figure 4**). As expected, food intake, locomotion, and arousal induced robust circadian fluctuations in EE, with peaks at night and minima during the day (p < 0.001). Maternal treatment had a significant overall effect on EE (p < 0.001): BPA offspring displayed increased EE associated with nighttime activity, whereas EE2 offspring showed consistently reduced values across both phases (**Figure 5**).

#### 3.2.4 Fuel utilization is differentially altered in the offspring of mothers exposed chronically to BPA or EE2

**Figure 6:**
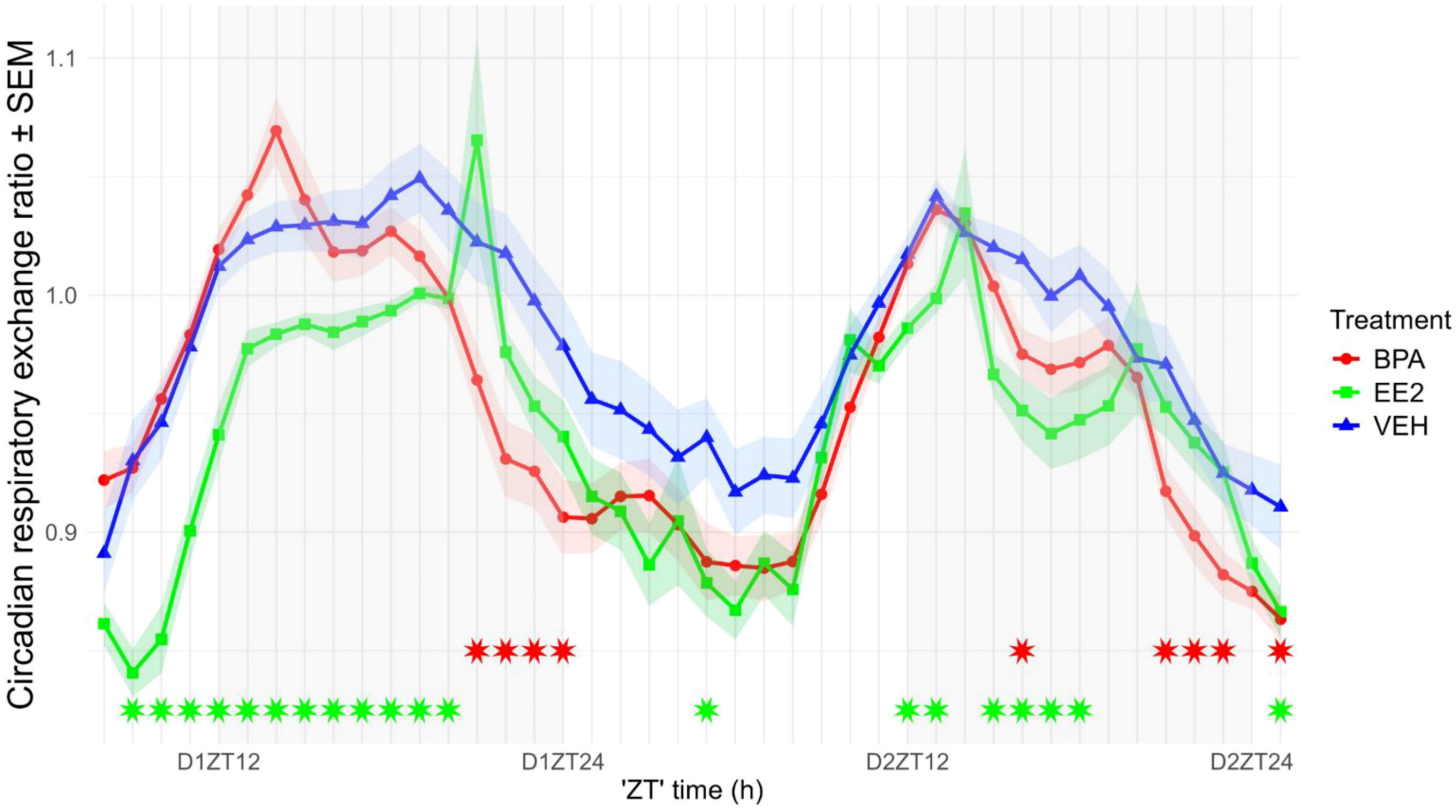
Circadian alterations in the respiratory exchange ratio (RER) of the offspring. RER values in EE2-exposed mice were primarily lower during the first part of the dark phase, whereas BPA-exposed mice showed decreased values mainly during its final period (ZT22–ZT24). Data are plotted as group means ± SEM. over time (recording started at 2:00 p.m., corresponding to ZT8). Lines and ribbons represent the group means and SEM, respectively. Red (BPA) and green (EE2) asterisks indicate ZT time points at which values differ significantly from those of VEH offspring. D1ZT12; Day1 Zeitgeber Time 12; BPA, Bisphenol A; EE2, 17α-Ethinylestradiol; VEH, vehicle.

The respiratory exchange ratio (the ratio between the amount of carbon dioxide produced- and oxygen used in metabolism) is the best estimate for judging substrates utilized during the day or night. Although RER values showed a clear oscillation with peak values during the night and a trough period during the day (p < 0.001); the values remained in the ±0.1 range of 1 indicating a preferential usage of carbohydrates as energy source. RER values of EE2 mice were significantly lower in both phases of day, whereas BPA mice displayed decreased values only during nighttime, mainly for its last period (ZT22-24), which suggests a slight transition in the metabolism towards using more fatty acids (**Figure 6**).

#### 3.2.5 24h fasting blood sugar levels were decreased in BPA offspring

**Figure 7:**
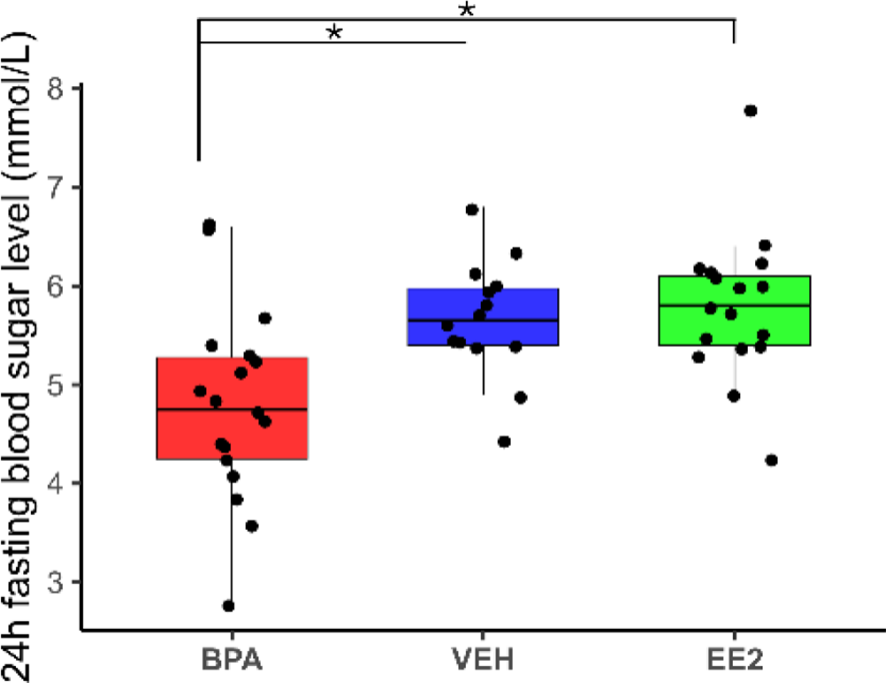
Twenty-four-hour fasting blood glucose levels. Blood samples were collected between ZT2 and ZT3. Box plots display the median, interquartile range (25^th^–75^th^ percentiles), and outliers for blood glucose levels in each treatment group, with points indicating individual mice. Blood glucose levels in BPA-exposed offspring were significantly lower than those in VEH or EE2 groups. BPA, Bisphenol A; EE2, 17^α^-Ethinylestradiol; VEH, vehicle.

Blood sugar level of the BPA offspring was significantly lower (p*_BPA-EE2_ = 0.00156, p_BPA-VEH_ = 0.0112*) than that of the VEH or EE2 mice *(BPA_fBGL_ 4.79 ± 0.28 mmol/L; VEH_fBGL_ 5.65 ± 0.16 mmol/L; EE2_fBGL_ 5.79 ± 0.18 mmol/L)* (**Figure 7**).

### 3.3 Litter-based analyses of the metabolic parameters of BPA, EE2 and VEH offspring

Offspring were obtained from six BPA-treated dams, four EE2-treated dams, and four control dams that received vehicle via osmotic minipumps. To determine whether offspring from the same litter could be considered statistically independent, litter effect analyses were performed for all metabolic and basic body composition parameters within each treatment group. Intra-class correlation coefficients (ICCs) and restricted likelihood ratio tests (RLRTs) were calculated for each parameter. Data were considered independent when ICC values were below 0.3 and/or when the inclusion of litter as a random effect did not significantly improve model fit (p > 0.05).

In most cases, these criteria were met, indicating negligible litter effects. However, significant litter effects were detected in the following cases: lean body mass in EE2 group; fasting blood glucose in BPA group; energy expenditure in BPA group at night 1 and day 2 and respiratory exchange ratio in BPA group at night 1, day 2 and in EE2 group at day 2. For these parameters, statistical analyses were repeated at the litter level.

Scheirer–Ray–Hare tests revealed significant main effects of treatment and time for both RER and energy expenditure. However, when treatment groups were compared at individual time points using Kruskal–Wallis tests with Benjamini–Hochberg correction, most time points did not retain the significant differences previously observed at the individual level. To visualize the relationship between litter-level and individual-level data, the same type of plots was generated for litter means (**Supplementary Figure S2**). The resulting patterns were consistent with those observed at the individual level, suggesting that the overall trends were preserved.

## 4 Discussion

The BPA dose used in this study (20 µg/kg bw/day) reflects the upper range of estimated human exposure prior to regulatory changes in the early 2000s, when the Tolerable Daily Intake (TDI) was 50 µg/kg/day. With growing evidence of BPA’s endocrine-disrupting and developmental effects, the TDI was reduced in Europe—first to 4 µg/kg/day in 2015, and more recently to 0.2 ng/kg/day in 2023. Despite these efforts, current average BPA intake remains around 0.01–0.05 µg/kg/day, with historically higher levels in infants. Given BPA’s potential role in the etiology of modern metabolic disorders, applying this dose in a mouse model remains relevant and scientifically justified.

To enable a comparison with another estrogenic compound, the synthetic estrogen EE2 was also administered to pregnant and lactating dams. Given that EE2 binds estrogen receptors (ERα and ERβ) with binding affinity hundreds to thousands of times stronger than BPA, a proportionally lower dose (0.01 µg/kg/day) was selected. This dose is 10 times lower than the environmentally relevant exposure used by Derouiche et al. (18) yet still within the upper range found in heavily polluted environments. Although typical EE2 concentrations in drinking water are below 10 ng/L, even such low levels may accumulate and produce developmental effects—particularly in fetuses and neonates.

In mice, circadian energy expenditure is determined by several components, including resting EE, diet-induced thermogenesis, physical activity, and arousal-associated EE. Results indicated that BPA mice not only eat less than their control counterparts but burn more calories by exhibiting higher resting EE and more activity in Z direction. The increase in rearing activity was particularly high by the onset of night causing a significant elevation in energy expenditure. The increased rearing activity in the first part of the night phase agrees with the observation of Nesan et al. (19), showing increased wheel-running activity in the same period of the objective and subjective night. It is worth noting that the locomotion of the current experimental animals in X-Y direction was not different from controls. The observed increase in vertical activity may reflect lower anxiety-like behavior, aligning with open field and social interaction tests by Nesan et al (19)., which indicated increased exploration and sociability in BPA-exposed offspring. The reduced food intake of BPA-exposed animals likely contributed to the decreased respiratory exchange ratio (RER) observed late at night, suggesting a temporary shift from carbohydrate to fat metabolism. These metabolic shifts could account for the lower fat mass and fasting blood glucose levels. Collectively, these results (based on individual-level data) indicate that perinatal BPA exposure is associated with a hyperactive, lean phenotype. Because the individuals examined were littermates, we accounted for litter effects in our analyses. At the litter level, some comparisons did not reach statistical significance despite significant differences at the individual level, although the overall trends remained consistent. This suggests that the lack of statistical significance likely reflects the limited sample size and resulting reduction in statistical power rather than the absence of a true biological effect. The fact that litter effects emerged only for a subset of metabolic and body composition parameters, while remaining negligible for most others, suggests that the treatments may have influenced not only the mean level of these traits but also the degree of similarity among littermates. In other words, treatment-dependent shifts in within-litter variability may underlie the selective appearance of litter effects.

Our model used mice on a C57BL/6J background, a strain known for its metabolic susceptibility, particularly under high-fat or high-sugar diets. The use of a standard diet in the current study may partially explain the reduced fat and body mass seen in BPA offspring. This idea is supported by Feighan et al. (11), who found that gestational BPA had anorexigenic effects in females on a standard diet but obesogenic effects when animals were fed high-fat chow. Interestingly, male offspring in that study showed no differences in weight, despite increased physical activity and unchanged caloric intake—an unexpected finding, given the energy demands of elevated activity (19). The absence of female offspring in our study precludes direct comparison of perinatal BPA and EE2 effects across sexes, including potential differences in circadian patterns of metabolic parameters.

In our study, BPA and EE2 offspring differed in several metabolic parameters. EE2-exposed animals showed lean body mass, fat mass, and fasting glucose levels like controls. Their activity levels across all axes of movement were also unaltered, suggesting no change in anxiety-like behavior, contrasting with prior findings (18). Like BPA offspring, EE2 animals consumed less food than controls. However, their reduced caloric intake was compensated by a corresponding decrease in energy expenditure throughout the light-dark cycle. This balance likely explains the lower RER values observed in the first part of the night phase, indicative of increased fat utilization.

Differences in receptor binding profiles may underlie the distinct outcomes observed between BPA and EE2 offspring. Although both compounds bind estrogen receptors, EE2 does so with much greater affinity and specificity, while BPA interacts with a broader array of receptors—including estrogen-related receptor γ, androgen, peroxisome proliferator-activated receptors, thyroid, and aryl hydrocarbon receptors (20, 21). A limitation of our study was the lack of body length measurements in the offspring. Thus, it remains uncertain whether BPA-exposed animals, besides being leaner, were also shorter. BPA offspring showed reduced lean and fat mass and a lower fat mass–to–lean body mass ratio, indicating a proportionally leaner phenotype. Without data on body length or growth hormone (GH) levels, we cannot exclude the possibility that BPA also affected somatic growth. Such effects would be consistent with disruption of endocrine pathways, including thyroid hormone and GH signaling, which may underlie the observed reductions in body mass components.

Both BPA and EE2 can influence the development of the suprachiasmatic nucleus (SCN), the brain’s central circadian pacemaker, which expresses high levels of ERβ, particularly in vasopressin (AVP) neurons (22). Perinatal exposure to endocrine disruptors like BPA has been shown to alter the number, morphology, and connectivity of SCN AVP neurons (11), potentially affecting their interactions with hunger-regulating AgRP/NPY neurons in the arcuate nucleus (23) and CRH neurons in the paraventricular nucleus (PVN) (24) or the dorsomedial nucleus (DMN) influencing the daily rhythm of feeding (25). Such circuit-level changes could explain the reduced food intake and altered metabolic rhythms observed in BPA- and EE2-exposed offspring. In addition to their effects on central circuits, BPA - and potentially EE2 via estrogen receptor signaling - might act directly on peripheral circadian oscillators. Animal studies have shown that developmental BPA exposure can alter expression of clock genes (e.g., Per2) in the liver (26). Although direct data for EE2 in peripheral tissue clocks are limited, estrogenic modulation of clock genes in metabolic organs has been documented (27). Such perturbations could lead to intrinsic timing disruption in metabolic tissues, possibly independent of SCN control. Over time, this internal desynchrony between central and peripheral rhythms might contribute to long-term impairments in feeding behavior, energy expenditure, and metabolic homeostasis in exposed offspring – a model that remains to be directly tested. Therefore, future studies are required to investigate not only clock gene expression, but also the coherence and coupling strength of the entire circadian system following early-life exposure to endocrine disruptors.

In conclusion, the current findings suggest that perinatal exposure to environmental doses of BPA or EE2 via the maternal route induces ontogenetic changes that result in altered, albeit subtly different, circadian regulation of energy homeostasis in male offspring. These differences likely stem from distinct yet partially overlapping mechanisms of action. The cumulative effect of early-life exposure to such endocrine-disrupting chemicals may predispose individuals to long-term metabolic disturbances. Such individuals may become particularly vulnerable to modern circadian stressors (shift work, irregular schedules, artificial light at night). Therefore, continued efforts to reduce environmental levels of these compounds are critical to minimizing developmental exposure and mitigating associated health risks.

## Data Availability

The original contributions presented in the study are included in the article/**Supplementary Material**. Further inquiries can be directed to the corresponding authors.

## Supplementary material

Supplementary Figure 1 Cumulative food intake of the offspring recorded over 42 hours (not normalized to lean body mass). BPA- and EE2-exposed offspring showed reduced food consumption compared with VEH controls. Data are plotted as group means ± s.e.m. over time (recording started at 2:00 p.m., corresponding to ZT8); grey bars indicate the dark phase (ZT12–ZT24). Lines and ribbons represent the group means and s.e.m., respectively. D1ZT12; Day1 Zeitgeber Time 12; BPA, Bisphenol A; EE2, 17α-Ethinylestradiol; VEH, vehicle.

Supplementary Figure 2 Litter mean ± s.e.m. values are shown for lean body mass, 24 h fasting blood glucose levels, energy expenditure, and respiratory exchange ratio in offspring from BPA (n = 21), VEH (n = 14), and EE2 (n = 17) groups. Box plots and line graphs with ribbons demonstrate similar patterns of differences in lean body mass, 24 h fasting blood glucose, and circadian alterations in energy expenditure and respiratory exchange ratio as those observed when individual animal values were analyzed. D1ZT12; Day1 Zeitgeber Time 12; BPA, Bisphenol A; EE2, 17α-Ethinylestradiol; VEH, vehicle.

## References

[1] López M Tena-Sempere M . Estrogens and the control of energy homeostasis: a brain perspective. Trends Endocrinol Metab. (2015) 26:411–21. doi: 10.1016/j.tem.2015.06.003, PMID: 26126705

[2] Singleton DW Khan SA . Xenoestrogen exposure and mechanisms of endocrine disruption. Front Biosci. (2003) 8:s110–8. doi: 10.2741/1010, PMID: 12456297

[3] E. Commission . Commission Regulation (EU) 2024/3190 of 19 December 2024 on the use of bisphenol A (BPA) and other bisphenols and bisphenol derivatives …, amending Regulation (EU) No 10/2011 and repealing Regulation (EU) 2018/213. Luxembourg: Publications Office of the European Union, Luxembourg (2024).

[4] Crews D Gore AC . Life imprints: living in a contaminated world. Environ Health Perspect. (2011) 119:1208–10. doi: 10.1289/ehp.1103451, PMID: 21571618 PMC3230404

[5] Caldwell DJ Mastrocco F Nowak E Johnston J Yekel H Pfeiffer D . An assessment of potential exposure and risk from estrogens in drinking water. Environ Health Perspect. (2010) 118:338–44. doi: 10.1289/ehp.0900654, PMID: 20194073 PMC2854760

[6] Takahashi O Oishi S . Disposition of orally administered 2,2-Bis(4-hydroxyphenyl)propane (Bisphenol A) in pregnant rats and the placental transfer to fetuses. Environ Health Perspect. (2000) 108:931–5. doi: 10.1289/ehp.00108931, PMID: 11049811 PMC1240124

[7] Mendonca K Hauser R Calafat AM Arbuckle TE Duty SM . Bisphenol A concentrations in maternal breast milk and infant urine. Int Arch Occup Environ Health. (2014) 87:13–20. doi: 10.1007/s00420-012-0834-9, PMID: 23212895 PMC4381877

[8] Frye CA Bo E Calamandrei G Calzà L Dessì-Fulgheri F Fernández M . Endocrine disrupters: a review of some sources, effects, and mechanisms of actions on behaviour and neuroendocrine systems. J Neuroendocrinol. (2012) 24:144–59. doi: 10.1111/j.1365-2826.2011.02229.x, PMID: 21951193 PMC3245362

[9] Sheehan DM Branham WS . Dissociation of estrogen-induced uterine growth and ornithine decarboxylase activity in the postnatal rat. Teratog Carcinog Mutagen. (1987) 7:411–22. doi: 10.1002/tcm.1770070408, PMID: 2442828

[10] Milligan SR Khan O Nash M . Competitive binding of xenobiotic oestrogens to rat alpha-fetoprotein and to sex steroid binding proteins in human and rainbow trout (Oncorhynchus mykiss) plasma. Gen Comp Endocrinol. (1998) 112:89–95. doi: 10.1006/gcen.1998.7146, PMID: 9748407

[11] Feighan KM Nesan D Kurrasch DM . Gestational bisphenol A exposure alters energy homeostasis and adult hypothalamic neurogenesis in female mice. Sci Rep. (2024) 14:16082. doi: 10.1038/s41598-024-66726-2, PMID: 38992091 PMC11239822

[12] Liu J Yu P Qian W Li Y Zhao J Huan F . Perinatal bisphenol A exposure and adult glucose homeostasis: identifying critical windows of exposure. PLoS One. (2013) 8:e64143. doi: 10.1371/journal.pone.0064143, PMID: 23675523 PMC3651242

[13] Mackay H Patterson ZR Khazall R Patel S Tsirlin D Abizaid A . Organizational effects of perinatal exposure to bisphenol-A and diethylstilbestrol on arcuate nucleus circuitry controlling food intake and energy expenditure in male and female CD-1 mice. Endocrinology. (2013) 154:1465–75. doi: 10.1210/en.2012-2044, PMID: 23493373

[14] Labaronne E Pinteur C Vega N Pesenti S Julien B Meugnier-Fouilloux E . Low-dose pollutant mixture triggers metabolic disturbances in female mice leading to common and specific features as compared to a high-fat diet. J Nutr Biochem. (2017) 45:83–93. doi: 10.1016/j.jnutbio.2017.04.001, PMID: 28433925

[15] Suter KJ Song WJ Sampson TL Wuarin JP Saunders JT Dudek FE . Genetic targeting of green fluorescent protein to gonadotropin-releasing hormone neurons: characterization of whole-cell electrophysiological properties and morphology. Endocrinology. (2000) 141:412–9. doi: 10.1210/endo.141.1.7279, PMID: 10614664

[16] Bingel AS Schwartz NB . Timing of LH release and ovulation in the post partum mouse. J Reprod Fertil. (1969) 19:231–7. doi: 10.1530/jrf.0.0190231, PMID: 5793459

[17] Weir JB . New methods for calculating metabolic rate with special reference to protein metabolism. J Physiol. (1949) 109:1–9. doi: 10.1113/jphysiol.1949.sp004363, PMID: 15394301 PMC1392602

[18] Derouiche L Keller M Martini M Duittoz AH Pillon D . Developmental exposure to ethinylestradiol affects reproductive physiology, the GnRH neuroendocrine network and behaviors in female mouse. Front Neurosci. (2015) 9:463. doi: 10.3389/fnins.2015.00463, PMID: 26696819 PMC4673314

[19] Nesan D Feighan KM Antle MC Kurrasch DM . Gestational low-dose BPA exposure impacts suprachiasmatic nucleus neurogenesis and circadian activity with transgenerational effects. Sci Adv. (2021) 7:abd1159. doi: 10.1126/sciadv.abd1159, PMID: 34049886 PMC8163075

[20] Richter CA Birnbaum LS Farabollini F Newbold RR Rubin BS Talsness CE . *In vivo* effects of bisphenol A in laboratory rodent studies. Reprod Toxicol. (2007) 24:199–224. doi: 10.1016/j.reprotox.2007.06.004, PMID: 17683900 PMC2151845

[21] Stanojević M Sollner Dolenc M . Mechanisms of bisphenol A and its analogs as endocrine disruptors via nuclear receptors and related signaling pathways. Arch Toxicol. (2025) 99:2397–417. doi: 10.1007/s00204-025-04025-z, PMID: 40116906 PMC12185661

[22] Vida B Hrabovszky E Kalamatianos T Coen CW Liposits Z Kallo I . Oestrogen Receptor alpha and beta Immunoreactive Cells in the Suprachiasmatic Nucleus of Mice: Distribution, Sex Differences and Regulation by Gonadal Hormones. J Neuroendocrinol. (2008) 20:1270–7. doi: 10.1111/j.1365-2826.2008.01787.x, PMID: 18752649

[23] Mendez-Hernandez R Escobar C Buijs RM . Suprachiasmatic nucleus-arcuate nucleus axis: interaction between time and metabolism essential for health. Obes (Silver Spring). (2020) 28 Suppl 1:S10–7. doi: 10.1002/oby.22774, PMID: 32538539

[24] Olejniczak I Campbell B Tsai YC Tyagarajan SK Albrecht U Ripperger JA . Suprachiasmatic to paraventricular nuclei interaction generates normal food searching rhythms in mice. Front Physiol. (2022) 13:909795. doi: 10.3389/fphys.2022.909795, PMID: 36277219 PMC9582613

[25] Starnes AN Jones JR . Inputs and outputs of the mammalian circadian clock. Biol (Basel). (2023) 12:508. doi: 10.3390/biology12040508, PMID: 37106709 PMC10136320

[26] Motta G Thangaraj SV Padmanabhan V . Developmental programming: impact of prenatal exposure to bisphenol A on senescence and circadian mediators in the liver of sheep. Toxics. (2023) 12:15. doi: 10.3390/toxics12010015, PMID: 38250971 PMC10818936

[27] Alvord VM Kantra EJ Pendergast JS . Estrogens and the circadian system. Semin Cell Dev Biol. (2022) 126:56–65. doi: 10.1016/j.semcdb.2021.04.010, PMID: 33975754 PMC8573061

