# Supplementary figures and images for: Metabolic consequences of perinatal bisphenol A and 17α-Ethinylestradiol exposure manifest in circadian alterations of energy homeostasis in adult male mice
Source: Front Endocrinol (Lausanne). 2026 Jan 6;16:1706909. doi: 10.3389/fendo.2025.1706909 (PMC12815843; doi:10.3389/fendo.2025.1706909)

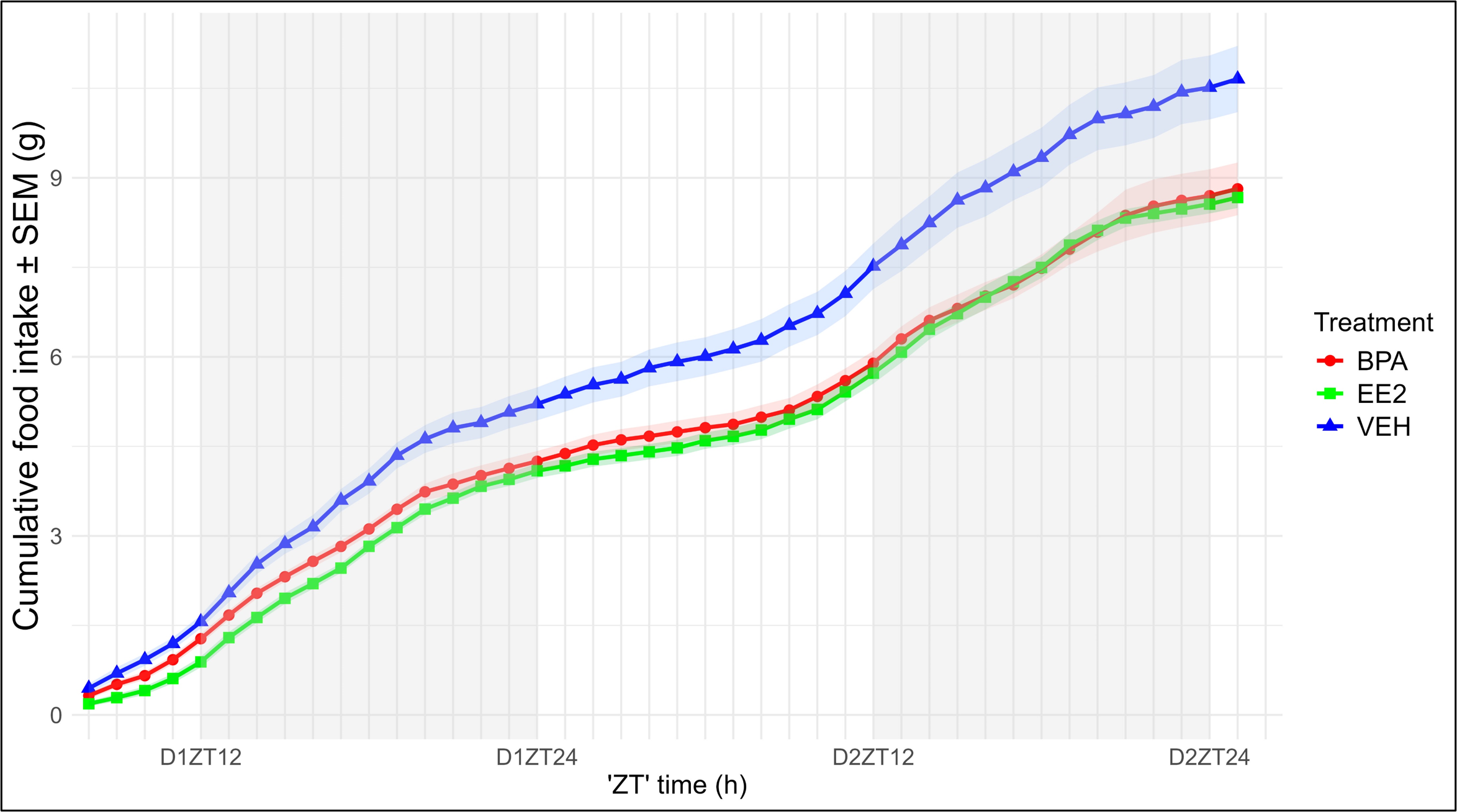

Supplement: Supplementary Figure 1 — Cumulative food intake of the offspring recorded over 42 hours (not normalized to lean body mass). BPA- and EE2-exposed offspring showed reduced food consumption compared with VEH controls. Data are plotted as group means ± s.e.m. over time (recording started at 2:00 p.m., corresponding to ZT8); grey bars indicate the dark phase (ZT12–ZT24). Lines and ribbons represent the group means and s.e.m., respectively. D1ZT12; Day1 Zeitgeber Time 12; BPA, Bisphenol A; EE2, 17α-Ethinylestradiol; VEH, vehicle. [file Image1.tif]

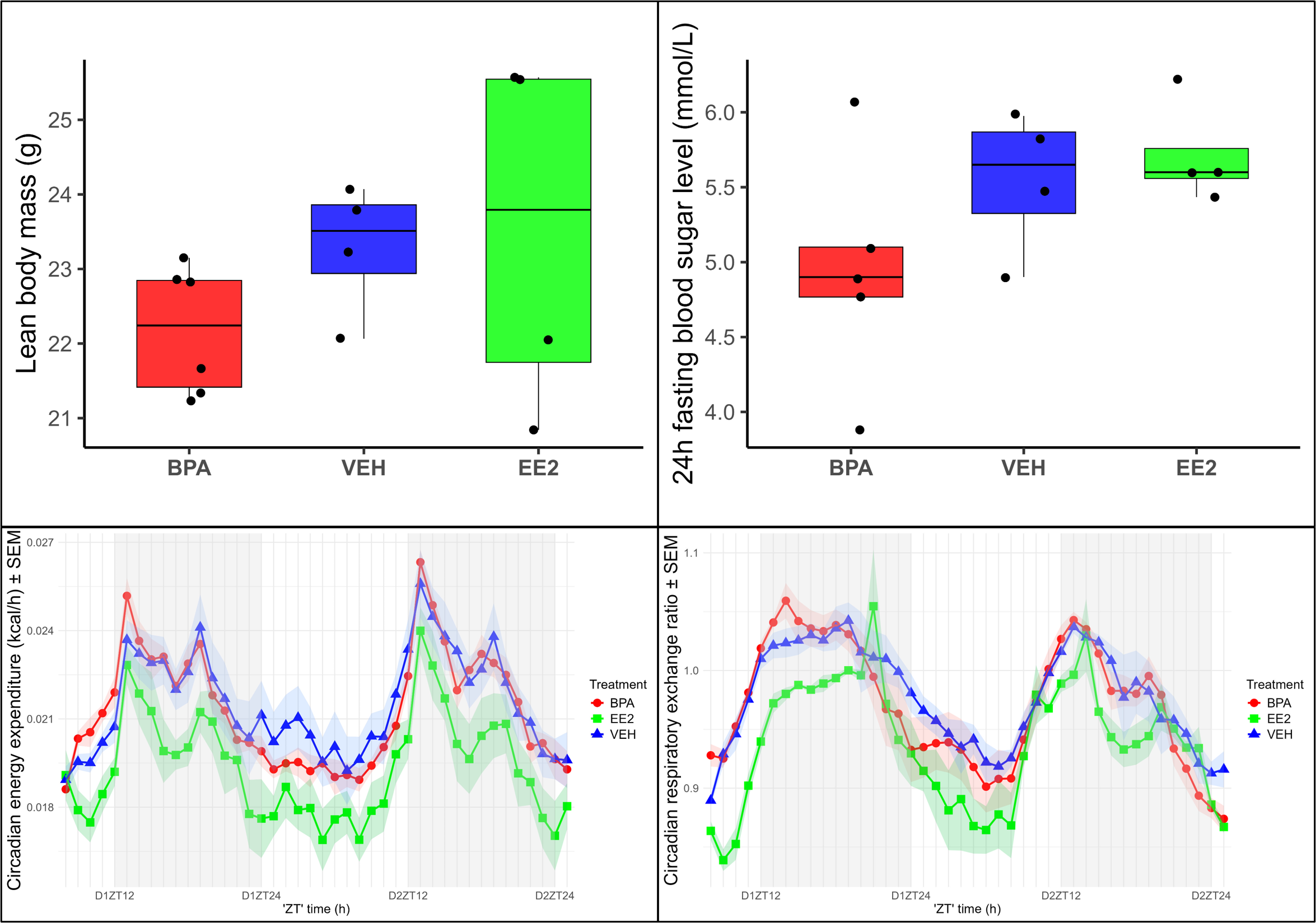

Supplement: Supplementary Figure 2 — Litter mean ± s.e.m. values are shown for lean body mass, 24 h fasting blood glucose levels, energy expenditure, and respiratory exchange ratio in offspring from BPA (n = 21), VEH (n = 14), and EE2 (n = 17) groups. Box plots and line graphs with ribbons demonstrate similar patterns of differences in lean body mass, 24 h fasting blood glucose, and circadian alterations in energy expenditure and respiratory exchange ratio as those observed when individual animal values were analyzed. D1ZT12; Day1 Zeitgeber Time 12; BPA, Bisphenol A; EE2, 17α-Ethinylestradiol; VEH, vehicle. [file Image2.tif]
