# Supplementary material for: Metabolic consequences of perinatal bisphenol A and 17α-Ethinylestradiol exposure manifest in circadian alterations of energy homeostasis in adult male mice
Source: Front Endocrinol (Lausanne). 2026 Jan 6;16:1706909. doi: 10.3389/fendo.2025.1706909 (PMC12815843; doi:10.3389/fendo.2025.1706909)
Supplement: Supplementary file 3 [file SupplementaryFile1.pdf]

S095

Approuvé le 12 nov-2019
par Dr. Gelania Goumbas
visa

ssniff

Mouse Breeding

fortified (autoclavable / for γ-irradiation)

Complete feed for mice – breeding (R/S elevage)

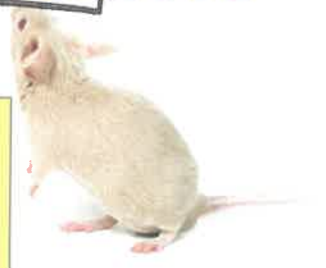

Description

The diet has been designed for the breeding of mice. The feed is characterized by a high energy density (supplementation of vegetable oil), a medium-high protein content with an excellent protein quality (amino acid composition), which makes this diet also suitable for the breeding of some transgenic mouse lines.

The diet is intended for ad libitum intake. The animals should have free access to fresh water

**Gross Energy (GE)**	**17.3**	**MJ/kg**
**Metabolizable Energy (ME)** [1]	**14.2**	**MJ/kg**

[1] = ME calculated according to the pig formula, Annex 4 of the German feed regulation

Physiological fuel value = 3445 kcal/kg

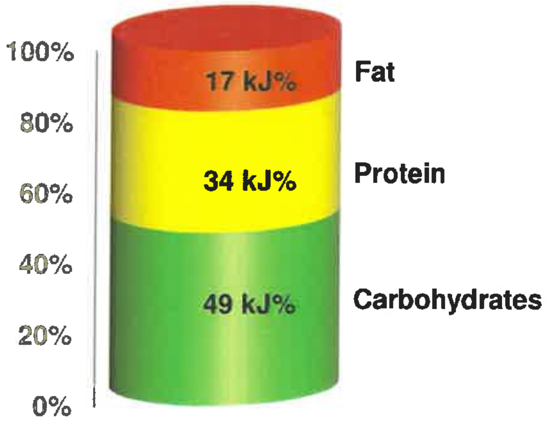

Crude Nutrients	[%]
Crude protein (N x 6.25)	21.0
Crude fat	7.0
Crude fibre	4.0
Crude ash	6.2
Starch	33.3
Sugar	4.6
N free extracts	49.4

Minerals	[%]
Calcium	1.10
Phosphorus	0.80
Ca / P	1.38 : 1
Sodium	0.25
Magnesium	0.20
Potassium	0.94

Fatty acids	[%]
C 12:0	—
C 14:0	0.02
C 16:0	0.80
C 18:0	0.26
C 20:0	0.03
C 16:1	0.04
C 18:1	1.68
C 18:2	3.73
C 18:3	0.46
C 20:5	—
C 22:6	—

Amino acids	[%]
Lysine	1.66
Methionine	0.91
Cystine	0.38
Met+Cys	1.29
Threonine	0.76
Tryptophan	0.26
Arginine	1.27
Histidine	0.51
Valine	0.98
Isoleucine	0.89
Leucine	1.57
Phenylalanine	0.99
Phe+Tyr	1.69
Glycine	0.88
Glutamic acid	4.38
Aspartic acid	1.99
Proline	1.41
Serine	1.09
Alanine	0.96

Vitamins	per kg	
Vitamin A	25,000	IU
Vitamin D_3	1,500	IU
Vitamin E	135	mg
Vitamin K (as MNB)	80	mg
Thiamine (B_1)	85	mg
Riboflavin (B_2)	32	mg
Pyridoxine (B_6)	31	mg
Cobalamin (B_{12})	150	µg
Nicotinic acid	140	mg
Pantothenic acid	59	mg
Folic acid	10	mg
Biotin	690	µg
Choline chloride	3,370	mg

Trace elements	per kg	
Iron	176	mg
Manganese	64	mg
Zinc	89	mg
Copper	16	mg
Iodine	2.2	mg
Selenium	0.3	mg

Dietary composition

descending order of feedingstuffs (FMV)

Grain and grain products, oil seed products, minerals, vegetable oils, brewer's yeast, amino acids, vitamins, trace elements

- Free from animal products / milk proteins -

Main products		Bag size
S8189-S094	10 mm, autoclavable	5 kg
S8189-S095	10 mm, autoclavable	25 kg
S8189-S095N	10 mm, autoclav., low hardness	25 kg
S8189-S096	10 mm, autoclavable	10 kg
S8189-S894	10 mm, γ-irradiated 40 kGy	5 kg vacuum
V1720/V1724	Meal / 10 mm	10 kg
V1724-300	10 mm, autoclavable	10 kg

Production and Sale
ssniff Spezialdiäten GmbH
Phone: +49-(0)2921-9658-0
Fax: +49-(0)2921-9658-40
E-Mail mail@ssniff.de
www.ssniff.de / www.ssniff.com

Status: 07/2016 & 11/2019 (addition of product codes)
